# Automated Multi-Class Classification of Retinal Pathologies: A Deep Learning Approach to Unified Ophthalmic Screening

**DOI:** 10.3390/diagnostics15212745

**Published:** 2025-10-29

**Authors:** Uğur Şevik, Onur Mutlu

**Affiliations:** 1 Department of Computer Science, Faculty of Science, Karadeniz Technical University, Ortahisar, Trabzon 61080, Türkiye; onurmutlu@ktu.edu.tr; 2 Retina R&D Software and Engineering Services Ltd., Trabzon Teknokent, Trabzon 61080, Türkiye

**Keywords:** deep learning, fundus photography, multi-class classification, computer-aided diagnosis, YOLO

## Abstract

**Background/Objectives**: The prevailing paradigm in ophthalmic AI involves siloed, single-disease models, which fails to address the complexity of differential diagnosis in clinical practice. This study aimed to develop and validate a unified deep learning framework for the automated multi-class classification of a wide spectrum of retinal pathologies from fundus photographs, moving beyond the single-disease paradigm to create a comprehensive screening tool. **Methods**: A publicly available dataset was manually curated by an ophthalmologist, resulting in 1841 images across nine classes, including Diabetic Retinopathy, Glaucoma, and Healthy retinas. After extensive data augmentation to mitigate class imbalance, three pre-trained CNN architectures (ResNet-152, EfficientNetV2, and a YOLOv11-based classifier) were comparatively evaluated. The models were trained using transfer learning and their performance was assessed on an independent test set using accuracy, macro-averaged F1-score, and Area Under the Curve (AUC). **Results**: The YOLOv11-based classifier demonstrated superior performance over the other architectures on the validation set. On the final independent test set, it achieved a robust overall accuracy of 0.861 and a macro-averaged F1-score of 0.861. The model yielded a validation set AUC of 0.961, which was statistically superior to both ResNet-152 (*p* < 0.001) and EfficientNetV2 (*p* < 0.01) as confirmed by the DeLong test. **Conclusions**: A unified deep learning framework, leveraging a YOLOv11 backbone, can accurately classify nine distinct retinal conditions from a single fundus photograph. This holistic approach moves beyond the limitations of single-disease algorithms, offering considerable promise as a comprehensive AI-driven screening tool to augment clinical decision-making and enhance diagnostic efficiency in ophthalmology.

## 1. Introduction

Vision impairment and blindness represent significant global health challenges, affecting an estimated 2.2 billion people worldwide, with at least 1 billion of these cases being preventable or yet to be addressed [1]. A substantial portion of this burden is attributable to posterior segment diseases of the eye, which often progress silently until they reach advanced, irreversible stages. Among these, diabetic retinopathy and glaucoma are the leading causes of preventable blindness in working-age adults and the elderly, respectively [2,3]. Other vision-threatening conditions, such as retinal detachment, a critical ophthalmic emergency, and pathologic myopia, a condition of growing prevalence, further contribute to the global burden of sight loss [4,5]. The efficacy of interventions for these pathologies is critically dependent on early and accurate detection, making widespread screening programs an essential pillar of public health strategies to mitigate irreversible visual disability.

Color fundus photography is the primary modality for retinal screening and diagnosis. This non-invasive, cost-effective technique provides high-resolution digital images of the retina, optic nerve head, and retinal vasculature, enabling clinicians to identify and document a wide array of pathological features [6]. Its utility has been pivotal in the expansion of teleophthalmology programs, which aim to bridge the gap in specialist eye care for remote and underserved populations. However, the interpretation of fundus images is a manual, time-consuming process that relies heavily on the availability of highly trained ophthalmologists and graders. This dependency creates a significant bottleneck in large-scale screening efforts, and the inherent subjectivity in human interpretation can lead to inter-observer variability, potentially affecting diagnostic consistency [7].

To address these challenges, the field of medical imaging has witnessed a paradigm shift with the advent of artificial intelligence (AI), particularly deep learning. Convolutional Neural Networks (CNNs), a class of deep learning algorithms, have demonstrated remarkable performance in image recognition tasks, often matching or even exceeding human-level accuracy [8]. In ophthalmology, this has led to the development of highly successful automated systems for detecting specific diseases in fundus photographs. Landmark studies have validated deep learning models for screening diabetic retinopathy and diabetic macular edema with exceptionally high sensitivity and specificity [9,10]. Similar successes have been reported for glaucoma detection, age-related macular degeneration, and classification of retinopathy of prematurity, establishing artificial intelligence as a robust tool for single-disease screening paradigms [11,12].

However, despite these successes, the prevailing paradigm in ophthalmic artificial intelligence is the development of siloed, single-disease models. These systems, while powerful for targeted screening, are trained to answer narrow binary questions, such as the presence or absence of DR, and are blind to other pathologies. This does not reflect the complex reality of a clinical encounter, where a patient may present with one of several conditions or even coexisting diseases. A model trained only to detect glaucoma, for example, would fail to flag a swollen disc caused by life-threatening intracranial pressure, creating a false sense of security and a missed opportunity for urgent intervention [13]. The clinical need is not just for disease detection but also for differential diagnosis, a sophisticated process of distinguishing between conditions with similar signs. This task is made more complex by the high inter-class similarity and intra-class variability in retinal pathologies, posing a substantial technical challenge that binary classifiers are ill-equipped to handle [14].

To address these ongoing challenges, research in this area focuses not only on improving accuracy but also on identifying efficient architectures, such as the YOLO backbone, that can be more easily integrated into clinical workflows. While various Convolutional Neural Network (CNN) based approaches have shown promise [15], the continued need for more robust and generalizable models persists, especially for handling high morphological similarity between pathologies and class imbalances common in medical datasets.

Therefore, the central objective of this study was to move beyond the single-disease paradigm and develop a comprehensive diagnostic framework capable of automated multi-class classification for a wide spectrum of retinal conditions. Achieving this goal with high fidelity requires more than simply extending a binary model; it necessitates the use of innovative deep learning architectures and training strategies that can learn subtle discriminative features from complex image data [16]. The central hypothesis of this study is that by leveraging a state-of-the-art deep learning architecture enhanced with mechanisms to improve feature representation and handle the inherent challenges of multi-class imbalance, we can create a unified model that performs a holistic initial assessment of fundus photographs with a high degree of diagnostic accuracy. This study’s framework is designed to simultaneously classify nine clinically crucial categories: Diabetic Retinopathy, Glaucoma, Pathologic Myopia, Retinal Detachment, Central Serous Chorioretinopathy, Retinitis Pigmentosa, Disc Edema, Macular Scar, and Healthy fundi.

To address this critical gap, this study details the design, development, and rigorous validation of a novel deep learning framework for the automated classification of nine distinct retinal conditions from fundus photographs. By leveraging a state-of-the-art CNN architecture and evaluating it on a large, independent dataset, we demonstrated that a single, unified model can achieve high diagnostic accuracy in differentiating a wide spectrum of pathologies, ranging from common diseases to critical emergencies. This study represents a significant step towards a comprehensive, artificial intelligence-driven screening tool capable of enhancing diagnostic workflows and improving patient care in diverse clinical settings.

## 2. Materials and Methods

### 2.1. Study Dataset

This study utilized the publicly available “A dataset of color fundus images for the detection and classification of eye diseases” by Sharmin et al. [17], sourced from Mendeley Data [18]. To address concerns regarding data quality and duplication within the original dataset, a rigorous manual curation process was conducted.

**Figure 1:**
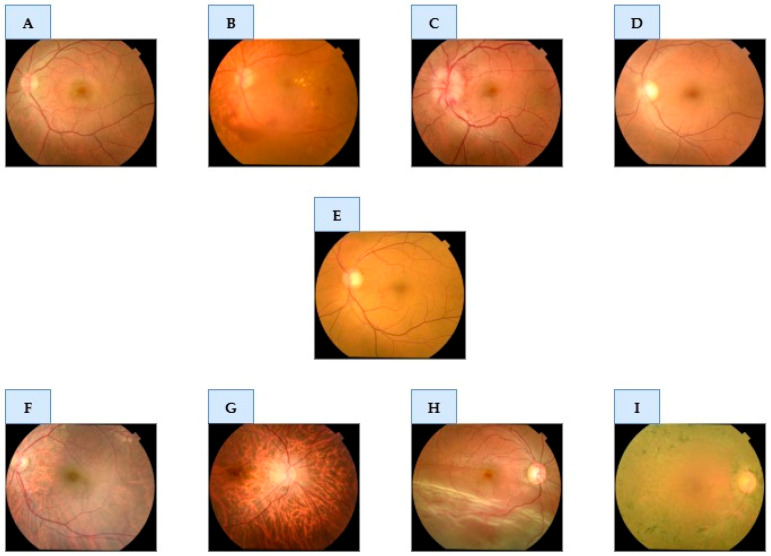
Exemplar color fundus photographs of each of the nine diagnostic classes evaluated in this study. The panels display: (**A**) Central Serous Chorioretinopathy; (**B**) Diabetic Retinopathy; (**C**) Disc Edema; (**D**) Glaucoma; (**E**) Healthy; (**F**) Macular Scar; (**G**) Pathologic Myopia; (**H**) Retinal Detachment; and (**I**) Retinitis Pigmentosa.

This process was supervised by an ophthalmologist with over twenty years of clinical experience in retinal image analysis. Each of the 5318 images was individually inspected to assess its quality and relevance to the designated diagnostic category. Images that were repetitive, of poor quality, or incorrectly labelled, and which did not clearly show the characteristics of the pathology, were excluded from the study. This meticulous review resulted in a refined and validated dataset of 1841 images. The final distribution of these images across the nine diagnostic classes is detailed in Table 1. Figure 1 provides representative examples from the curated dataset for each of the nine classes.

### 2.2. Ethical Statement

This study was conducted in accordance with the tenets of the Declaration of Helsinki. This study exclusively utilized the “Eye Disease Image Dataset” [17], a publicly available and fully anonymized dataset. As all patient-identifying information was removed by the original data curators prior to public release, this secondary analysis did not require separate institutional review board or ethics committee approval.

### 2.3. Data Preprocessing and Augmentation

A rigorous data preparation pipeline was implemented to ensure model robustness, optimize learning efficiency, and mitigate the risk of overfitting, comprising three primary stages: image preprocessing, dataset partitioning, and data augmentation. In the preprocessing stage, all images were resized to a uniform resolution of 224 × 224 pixels and their pixel values were normalized to a range of [0, 1].

Subsequently, the curated dataset of 1841 images was partitioned using stratified sampling to preserve the class distribution, creating a training set (70%; 1291 images), a validation set (15%; 276 images), and a test set (15%; 274 images). To enhance the model’s ability to generalize and to address the inherent class imbalance, the 1291 image training set was then subjected to a comprehensive data augmentation strategy. A suite of augmentation techniques was strategically applied to increase the sample count of under-represented classes and create a more balanced distribution. The augmentation techniques included the following.

- Random Rotations: Images were randomly rotated within a range of ±15 degrees.
- Horizontal Flipping: Images were randomly flipped horizontally.
- Random Zoom: A random zoom of up to 20% was applied to the images.
- Brightness and Contrast Adjustments: Brightness and contrast were randomly altered by up to 20%.
- Shift Augmentation: Images were randomly shifted both horizontally and vertically by up to 10% of their dimensions.

**Figure 2:**
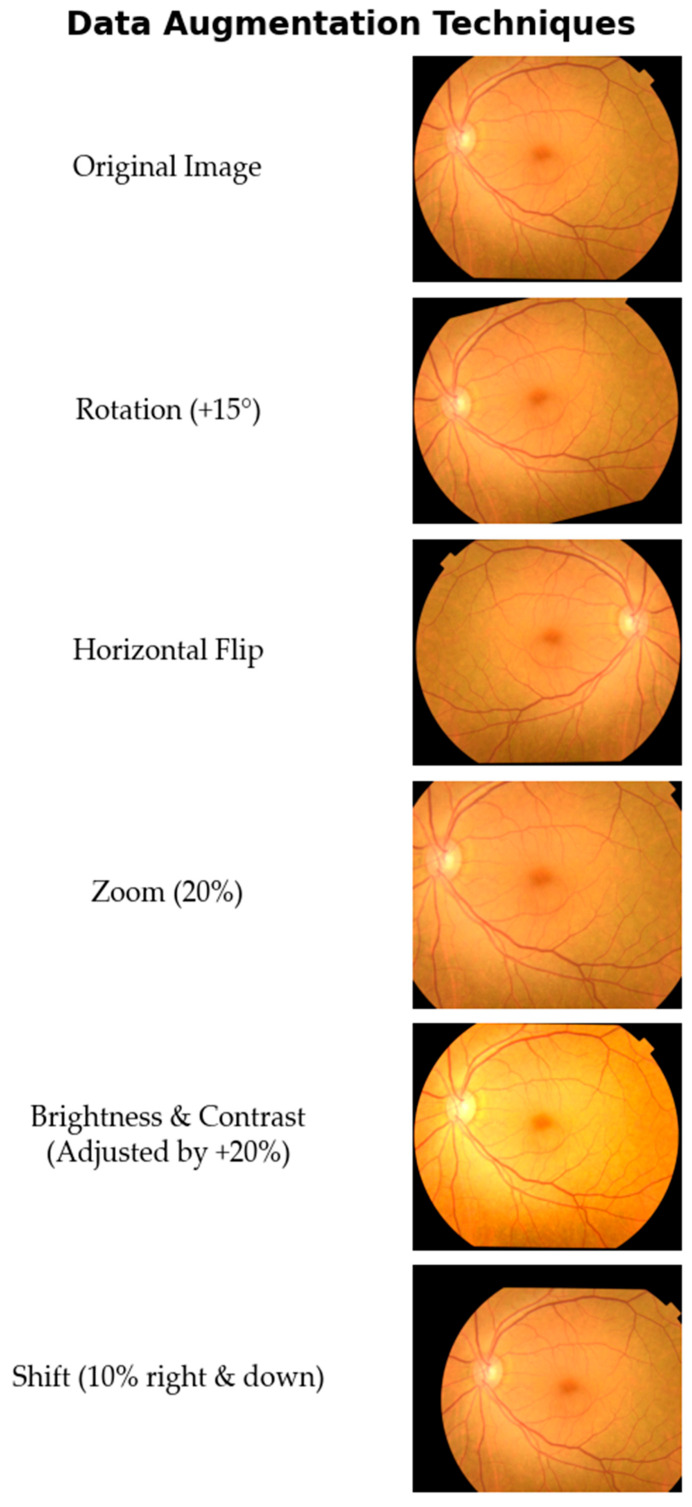
Visual examples of the data-augmentation techniques applied to a sample fundus image. Starting from the original image, a series of transformations were applied to generate synthetic training data, including rotation, horizontal flipping, zooming, brightness and contrast adjustment, and spatial shifting.

A visual demonstration of these augmentation techniques applied to a sample fundus image is shown in Figure 2.

The primary objective of applying data augmentation was to significantly expand the training dataset. By introducing a greater diversity of data into the training process, the goal was to enhance the model’s robustness and achieve higher performance. To this end, seven new synthetic images were generated from each original image in the training set by applying the rotation and shift techniques twice, while the other three techniques were applied once. As a result, the training set was expanded from 1291 to 10,328 images, creating a larger and more varied corpus instrumental for improving generalization and preventing overfitting.

### 2.4. Deep Learning Model Architecture

To address the multi-class classification task, we employed and compared three distinct deep learning architectures: ResNet-152, EfficientNetV2, and a classifier based on the YOLOv11 backbone. A cornerstone of our methodology was transfer learning, where each model was initialized with weights pre-trained on the ImageNet dataset [19]. This approach leverages a rich hierarchy of pre-learned visual features to accelerate convergence and enhance performance on specialized medical datasets.

Our first model was ResNet-152, a member of the Deep Residual Network family known for its proven robustness in medical imaging tasks [20]. ResNet’s fundamental innovation is the use of “residual” or “skip” connections, which mitigate the vanishing gradient problem in deep networks. This design allows for the successful training of very deep networks, such as 151 layers, enabling the model to learn complex and hierarchical features.

The second architecture was EfficientNetV2, a state-of-the-art model renowned for its balance between high accuracy and computational efficiency [21]. The EfficientNet family introduced a novel “compound scaling” method, which uniformly scales network depth, width, and resolution to achieve superior performance for a given computational cost. EfficientNetV2 further improves this with architectural optimizations like Fused-MBConv blocks, which enhance parameter efficiency and accelerate the training process.

As a third approach, we utilized the powerful convolutional backbone of the YOLOv11 model as a feature extractor. While the YOLO family is primarily known for real-time object detection, its underlying backbone is engineered to efficiently extract rich, salient features in a single pass [22]. We repurposed this backbone to leverage its optimized feature extraction power for differentiating the fine-grained pathological features in fundus images.

**Figure 3:**
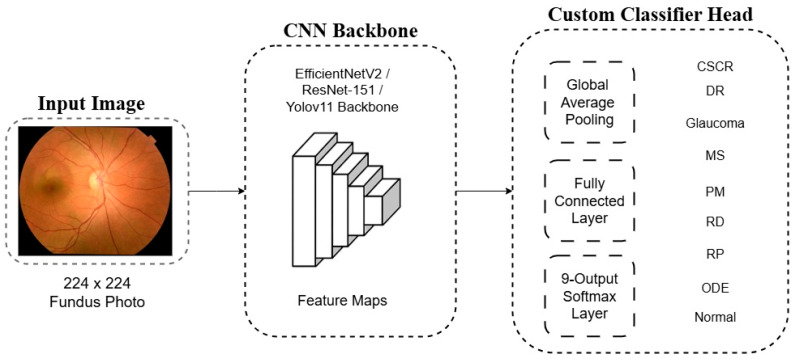
Architecture of the proposed deep learning model. A pre-trained CNN backbone extracts features that are passed to a custom classifier head for 9-class classification using a softmax output layer.

For each backbone, the final ImageNet-specific layer was removed and replaced with a custom classifier head tailored to our 9-class problem. This new head consists of a Global Average Pooling (GAP) layer, a fully connected layer, and a final softmax layer that outputs a probability distribution across the nine diagnostic classes. A schematic representation of this architectural adaptation is shown in Figure 3.

### 2.5. Model Training and Implementation

All models were implemented in Python (version 3.11) using the PyTorch library (version 2.5.1) with CUDA 12.6 acceleration. Experiments were conducted on a high-performance workstation equipped with an Intel Core i9-14900K CPU, 64 GB of DDR5 RAM, and an NVIDIA GeForce RTX 4090 GPU with 24 GB of VRAM.

The models were trained using the AdamW optimizer [23] and a categorical Cross-Entropy Loss function. The initial learning rate was set to 1 × 10^−4^, coupled with a ReduceLROnPlateau scheduler that reduced the learning rate by a factor of 10 if validation loss did not improve for 5 consecutive epochs. Training was performed with a batch size of 32 for a maximum of 100 epochs. An early stopping mechanism was implemented, halting the training if validation loss failed to improve for 15 consecutive epochs and saving the model weights from the epoch with the lowest validation loss for final evaluation.

### 2.6. Performance Evaluation and Statistical Analysis

The models’ diagnostic performance was evaluated on a strictly segregated, independent test set for an unbiased assessment of generalizability. A set of standard statistical metrics was computed, with formulas presented in Table 2. These metrics Accuracy, Precision, Recall, and F1-Score were derived from the four cardinal outcomes of the confusion matrix: true positives (TP), true negatives (TN), false positives (FP), and false negatives (FN). To account for class imbalance, the metrics were aggregated using a macro-average, which treats all classes equally.

To further assess performance, the Area Under the Receiver Operating Characteristic Curve (AUC) was computed using a one-vs-rest strategy to evaluate discriminative capability. A 9 × 9 confusion matrix was also generated to qualitatively analyze systematic inter-class confusions. All computations and visualizations were performed using the scikit-learn (1.7.0), Matplotlib (3.10.3), and Seaborn (0.13.2) libraries.

## 3. Results

The diagnostic performance of the three deep learning models was rigorously evaluated through a two-phase protocol. In the initial phase, a comparative assessment was conducted on the validation set to identify the optimal architecture and hyperparameters. Subsequently, the highest-performing model was subjected to a definitive evaluation on the independent test set, which remained entirely sequestered during training and validation to ensure an unbiased measure of generalizability.

### 3.1. Comparative Evaluation of Classification Models

**Figure 4:**
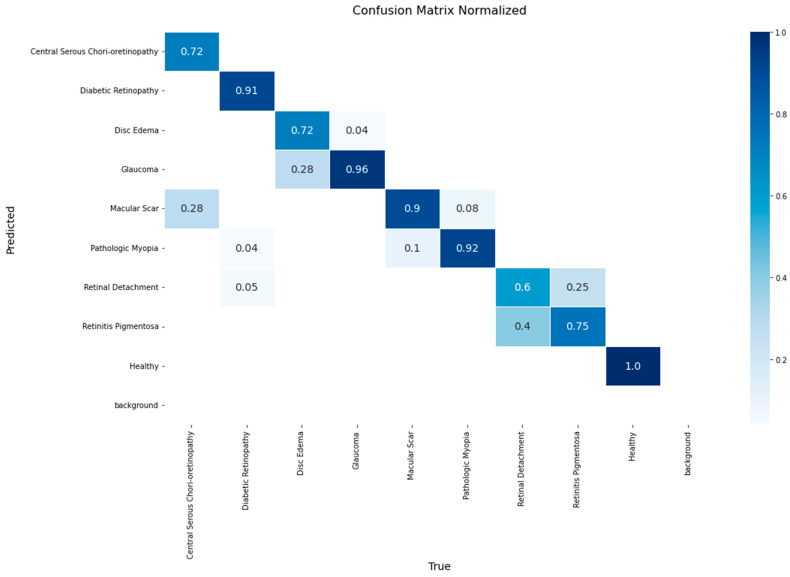
Normalized confusion matrix for the EfficientNetV2 model on the validation set.

The performance of the EfficientNetV2 model on the validation set is presented in the normalized confusion matrix in Figure 4. The model achieved a accuracy of 1.00 for the Healthy class, followed by Glaucoma at 0.96 and Pathologic Myopia at 0.92. The lowest accuracy values, 0.6, were observed in Retinal Detachment.

The matrix also reveals specific inter-class confusions. 28% of Disc Edema cases were misclassified as Glaucoma, and 28% of Macular Scar cases were misclassified as Central Serous Chorioretinopathy. Furthermore, 40% of Retinal Detachment instances were classified as Retinitis Pigmentosa.

**Figure 5:**
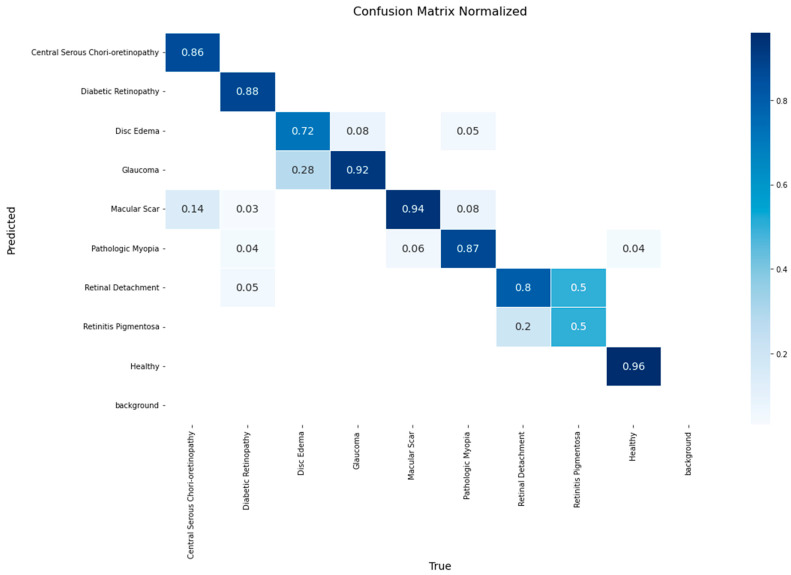
Normalized confusion matrix for the ResNet-152 model on the validation set.

Figure 5 presents the normalized confusion matrix for the ResNet-152 model on the validation set. High accuracy values were achieved for Healthy (0.96), Macular Scar (0.94), and Glaucoma (0.92) classes, whereas Retinitis Pigmentosa yielded the lowest recall (0.50). Notable confusions included 50% of Retinitis Pigmentosa cases misclassified as Retinal Detachment and 28% of Disc Edema cases as Glaucoma.

**Figure 6:**
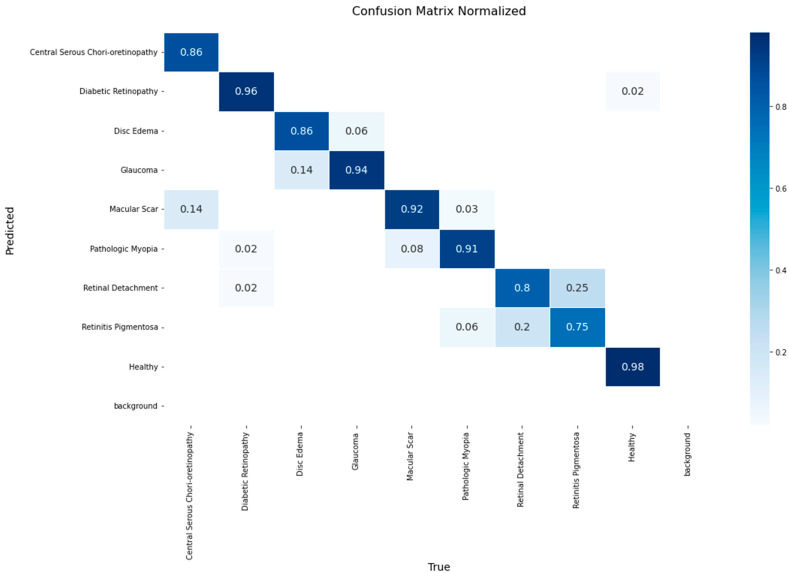
Normalized confusion matrix for the YOLOv11-based classifier on the validation set.

The YOLO-based classifier demonstrated a strong discriminative power, as detailed in the normalized confusion matrix shown in Figure 6. The model achieved exceptional accuracy rates for several classes, notably reaching 0.98 for the Healthy class, 0.96 for Diabetic Retinopathy, and 0.94 for Glaucoma.

Despite its high overall performance, specific inter-class confusions were observed. A notable confusion occurred between optic nerve head pathologies, where 14% of Disc Edema cases were misclassified as Glaucoma. Furthermore, 14% of Central Serous Chorioretinopathy cases were incorrectly classified as Macular Scar. The model also showed confusion in distinguishing Retinitis Pigmentosa, misclassifying 20% of its cases as Retinal Detachment.

For a quantitative comparison of the models’ diagnostic capabilities, overall accuracy and macro-averaged recall, precision, and F1-score were computed on the validation set, with the results summarized in Table 3. The analysis reveals that the YOLOv11-based classifier substantially outperformed the other architectures across all metrics, achieving a top accuracy of 0.887 and an F1-score of 0.889.

In comparison, EfficientNetV2 and ResNet-152 delivered competitive but lower performance. EfficientNetV2 registered an accuracy of 0.831 and an F1-score of 0.845, while ResNet-152 recorded an accuracy of 0.828 and an F1-score of 0.841. These quantitative results are consistent with the qualitative analysis of the confusion matrices, reinforcing the selection of the YOLOv11-based framework as the most robust and accurate model for this multi-class classification task.

**Figure 7:**
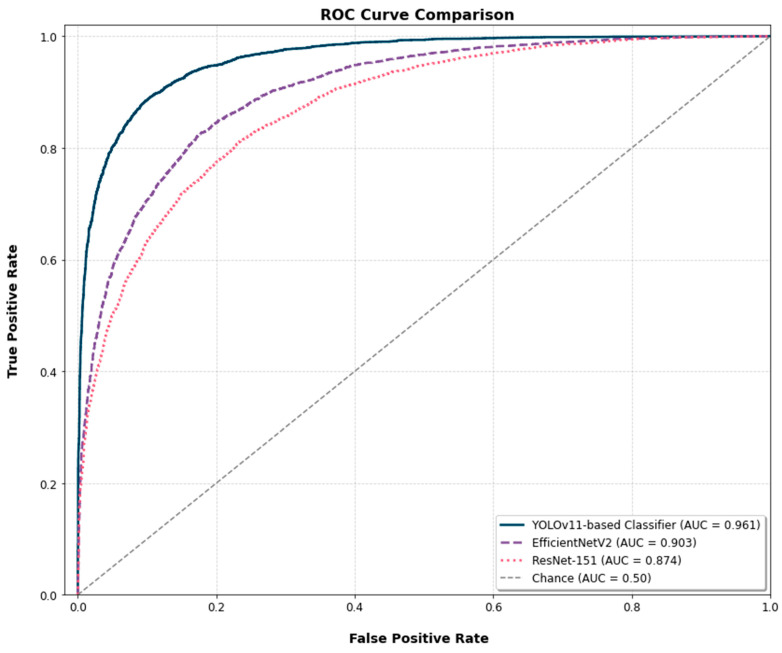
Comparison of ROC curves for the three models, with the Area Under the Curve (AUC) indicated for each. The dashed line represents the random classifier baseline (AUC = 0.50).

The Receiver Operating Characteristic (ROC) curves for the three models are presented for a comparative analysis of their diagnostic discrimination ability (Figure 7). The YOLOv11-based classifier achieved an Area Under the Curve (AUC) of 0.961. The EfficientNetV2 and ResNet-152 models yielded AUC scores of 0.903 and 0.874, respectively. To assess the statistical significance of these differences, the DeLong test was applied. The test confirmed that the AUC of the YOLOv11-based classifier was significantly higher than that of both ResNet-152 (*p* < 0.001) and EfficientNetV2 (*p* < 0.01).

### 3.2. Final Model Evaluation on the Test Set

To assess the generalization capability of the selected YOLOv11-based classifier, a final evaluation was conducted on the independent test set. On this unseen data, the model achieved a robust overall accuracy of 0.861, with a macro-averaged recall of 0.873 and precision of 0.861, confirming its ability to generalize effectively.

**Figure 8:**
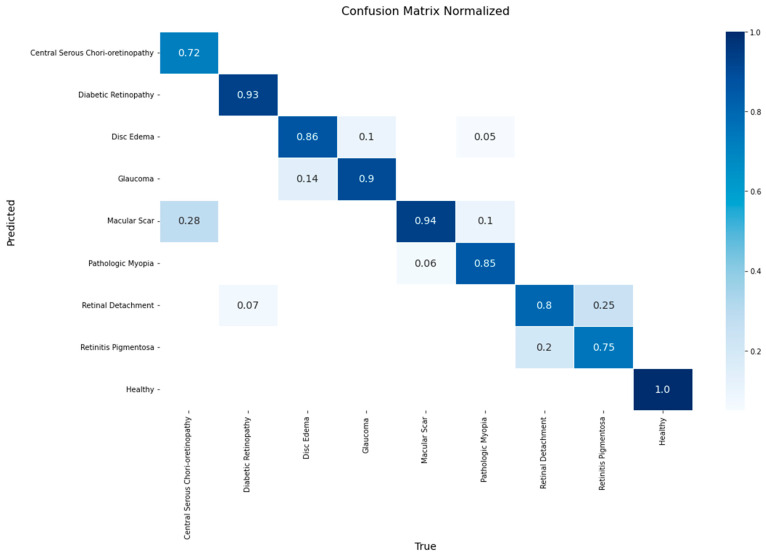
Normalized confusion matrix for the YOLOv11-based classifier on the test set.

A detailed breakdown of the model’s class-wise performance is presented in the confusion matrix in Figure 8. The model demonstrated high efficacy, achieving the highest accuracy rates for the Healthy (1.00), Macular Scar (0.94), and Diabetic Retinopathy (0.93) classes. It also showed robust performance for Glaucoma (0.90) and Disc Edema (0.86). However, the evaluation also highlighted specific diagnostic challenges, particularly in classes with lower recall rates such as Central Serous Chorioretinopathy (0.72) and Retinitis Pigmentosa (0.75). The most significant source of error was the confusion between macular pathologies, where 28% of Central Serous Chorioretinopathy cases were misclassified as Macular Scar. Furthermore, a notable confusion was observed between Retinitis Pigmentosa and Retinal Detachment, with 25% of the former’s cases being misclassified as the latter.

**Figure 9:**
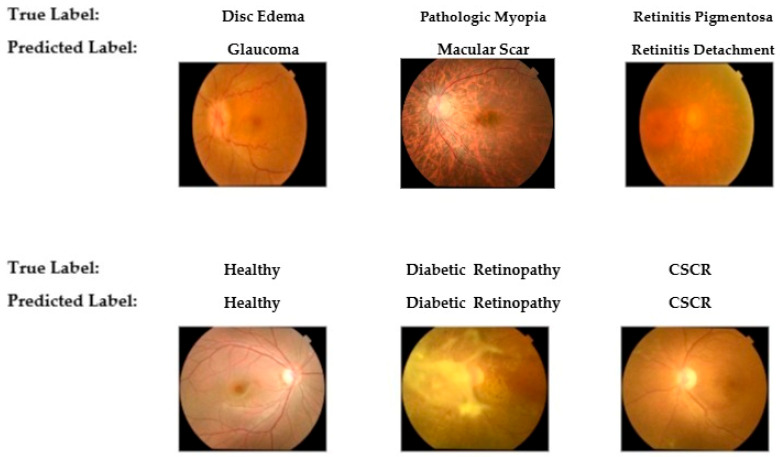
Representative examples of correct (**bottom row**) and incorrect (**top row**) classifications by the final YOLOv11-based model on the test set.

To provide qualitative insight into the model’s diagnostic behavior, Figure 9 presents a selection of classification examples from the test set. The bottom row illustrates the model’s successful classifications on cases with distinct features, such as Healthy, Diabetic Retinopathy, and Central Serous Chorioretinopathy (CSCR). Conversely, the top row displays misclassifications that occurred in cases with overlapping morphological features. These errors, including the confusion of Disc Edema with Glaucoma and Retinitis Pigmentosa with Retinal Detachment, visually corroborate the challenging diagnostic patterns identified in the confusion matrix.

## 4. Discussion

In this study, we developed and validated a deep learning framework designed to address the critical need for automated, multi-class classification of retinal pathologies from color fundus photographs. By systematically evaluating three distinct CNN architectures ResNet-152, EfficientNetV2, and a clasifier leveraging the potent feature extraction capabilities of a YOLOv11 backbone our findings unequivocally identified the YOLOv11-based model as the most effective for this complex diagnostic task. The selected model demonstrated robust generalization with a final accuracy of 0.861 on a strictly independent test set, a result underpinned by its excellent discriminative performance, as evidenced by an Area Under the Curve (AUC) of 0.961 on the validation set. This high level of performance not only substantiates our central hypothesis but also showcases that a single, unified model can transcend the inherent limitations of single-disease screening paradigms. Therefore, this work represents a significant step toward a clinically versatile AI tool capable of tackling the nuanced challenge of differential diagnosis in routine ophthalmic screening.

A key finding of this study was the robust performance of the YOLOv11-based classifier, which demonstrated high efficacy not only in its overall metrics but also in the identification of key pathologies. The model achieved exceptional accuracy rates on the test set for the Healthy (1.00), Macular Scar (0.94), and Diabetic Retinopathy (0.93) classes. The model’s macro-averaged F1-score of 0.889 on the validation set indicates robust performance. While direct metric comparisons between studies are challenging due to differing datasets and methodologies, these results can be contextualized against other multi-class classification frameworks. For instance, our F1-score is comparable to benchmarks reported by Yang and Yi (88.16%) [24] and Islam et al. (85%) [25]. However, this comparison must be interpreted with significant caution. As noted in our limitations, our model was trained on a curated dataset of well-defined pathologies, which may not reflect the full spectrum of disease severity encountered in the datasets used for other state-of-the-art models. Therefore, these figures are presented as contextual benchmarks rather than a direct claim of superior or competitive performance. While some advanced models integrating metadata have reported higher scores, such as the 94.11% F1-score by Deng and Ding [26], our framework’s strong performance is notable because it relies exclusively on pixel data from the fundus image, enhancing its potential for broader clinical applicability. This was achieved using a repurposed YOLOv11 backbone, suggesting that architectures designed for efficient object detection possess powerful feature extraction capabilities that are highly adept at identifying the subtle, localized indicators of retinal disease.

Despite its high overall accuracy, an analysis of the model’s misclassifications provides valuable insight into diagnostic challenges that mirror known difficulties in clinical ophthalmology. The most persistent confusion was observed between pathologies affecting the optic nerve head; on the validation set, 14% of Disc Edema cases were misclassified as Glaucoma. This specific error underscores a significant clinical challenge, as differentiating between glaucomatous optic neuropathy (GON) and non-glaucomatous optic neuropathies (NGONs) can be exceptionally difficult owing to overlapping features such as optic disc cupping. This distinction is so complex that dedicated deep learning frameworks have been developed specifically to address it, as demonstrated by Vali et al. [27]. Similarly, our model exhibited confusion between certain macular pathologies, misclassifying 28% of Central Serous Chorioretinopathy cases as Macular Scar on the test set. These findings highlight that as artificial intelligence systems advance from binary classification to complex differential diagnosis, the primary hurdle is resolving the ambiguity caused by the high inter-class similarity and intra-class variability inherent in retinal pathologies.

The diagnostic accuracy achieved in this study is attributable not only to the selection of an advanced network architecture but also to the implementation of a meticulous data preparation and augmentation pipeline. A primary challenge in developing robust medical AI models is the often-limited size and significant class imbalance of available datasets. Our strategy directly addressed this by employing a comprehensive suite of targeted data augmentation techniques, which expanded the training set from 1177 to 10,328 images. This process, particularly the strategic oversampling of minority classes, was instrumental in mitigating class-imbalance-related biases and preventing model overfitting. The importance of such rigorous data preparation is widely recognized; reviews of deep learning in medical imaging consistently highlight data augmentation as a cornerstone for improving model generalization. This reinforces the principle that for deep learning models to achieve clinical viability, the sophistication of the data strategy is as critical as the novelty of the network architecture itself.

Although the results of this study are promising, several limitations must be acknowledged. A primary limitation stems from the composition of the dataset used for training and validation. The manual curation process, while improving label accuracy, intentionally created a “clean” dataset consisting of high-quality images that represent each pathology with its most distinct and characteristic features. Consequently, the dataset predominantly features well-defined, significant pathologies and does not encompass the full spectrum of clinical presentation, most notably mild, subtle, or early-stage diseases. This well-defined data structure is a significant confounding factor, as it may have favored architectures, such as the YOLO backbone, that are highly adept at recognizing large and distinct patterns. Therefore, the model’s high performance must be interpreted with caution, as its capability to generalize to the more ambiguous and nuanced findings frequently encountered in a real-world clinical screening setting has not been assessed. Furthermore, the framework was developed and validated using a single public dataset from a specific geographic region. This reliance on a single source, compounded by the dataset’s bias towards clear-cut pathologies, limits the model’s proven generalizability. The model’s performance has not been externally validated on datasets from different ethnic populations, on images acquired with a variety of fundus cameras, or, critically, on clinical datasets that include a realistic distribution of disease severities. The potential for performance degradation when a model is applied to unseen data from different sources underscores the critical need for further validation to establish the model’s true robustness in diverse clinical settings.

Therefore, future work must build upon this foundation by addressing these limitations directly. First, the immediate next step is to rigorously validate the model’s performance and generalizability. This will necessitate testing on a wide range of external data, which should include not only diverse, multicenter, and multiethnic public datasets but also proprietary clinical data from our own institution to ensure real-world applicability. Second, future architectural refinements will focus on improving the model’s ability to discriminate between challenging pairs with high inter-class similarity, such as Glaucoma and Disc Edema. Third, to address the crucial need for model transparency in medical AI, we plan to integrate explainability techniques. Implementing methods such as Grad-CAM will be essential to visualize the model’s decision-making process, ensure it focuses on clinically relevant pathological features, and ultimately build trust among clinicians. Finally, looking toward clinical translation, prospective studies are required to assess how a comprehensive screening tool like this would integrate into diagnostic workflows and impact patient care. Nevertheless, the current study provides a robust starting point for developing a clinically deployable, comprehensive retinal screening tool.

## 5. Conclusions

This study successfully developed and validated a unified deep learning framework capable of classifying nine distinct and clinically significant retinal conditions from a single fundus photograph. Our comparative analysis identified a YOLOv11-based classifier as the most effective architecture, achieving a robust final accuracy of 0.861 on an independent test set and demonstrating high discriminative capability with an Area Under the Curve (AUC) of 0.961. The model showed a strong ability to classify this predefined set of pathologies, which includes high-prevalence diseases such as Diabetic Retinopathy and Glaucoma, as well as less common but critical findings like Disc Edema. This work provides compelling evidence that a single, holistic model can function as a comprehensive initial screening tool, presenting an approach that moves beyond the inherent limitations of single-disease algorithms. While external validation on diverse, multiethnic datasets is a crucial next step, the proposed framework holds considerable promise as an artificial intelligence-driven tool to augment clinical decision-making, enhance diagnostic efficiency, and ultimately improve patient outcomes in ophthalmology.

## Figures and Tables

**Table 1:** Distribution of images across nine diagnostic classes.

Class Number	Class Name	Number of Images
0	Central Serous Chorioretinopathy	48
1	Diabetic Retinopathy	389
2	Disc Edema	47
3	Glaucoma	463
4	Macular Scar	167
5	Pathologic Myopia	210
6	Retinal Detachment	32
7	Retinitis Pigmentosa	28
8	Healthy	457

**Table 2:** Definitions and formulas of the primary performance evaluation metrics.

Metric	Formula	Description
Accuracy	$\frac{TP+TN}{TP+TN+FP+FN}$	The proportion of all correct predictions among the total number of cases.
Precision	$\frac{TP}{TP+FP}$	The proportion of correct positive predictions among all positive predictions.
Recall	$\frac{TP}{TP+FN}$	The proportion of actual positives that were correctly identified.
F1-Score	$2\times \frac{Precision\times Recall}{Precision+Recall}$	The harmonic mean of Precision and Recall, providing a single score that balances both.

**Table 3:** Performance comparison of the deep learning models on the validation set, showing overall accuracy with macro-averaged recall, precision, and F1-score.

	Accuracy	Recall	Precision	F1-Score
EfficientNetV2	0.831	0.847	0.844	0.845
ResNet-152	0.828	0.841	0.842	0.841
YOLOv11-based Classifier	0.887	0.891	0.887	0.889

## Data Availability

The “Eye Disease Image Dataset” used to support the findings of this study is publicly available from the Mendeley Data repository at https://doi.org/10.17632/s9bfhswzjb.1, as referenced in [18].

## References

[1] Al-Fahdawi S., Al-Waisy A.S., Zeebaree D.Q., Qahwaji R., Natiq H., Mohammed M.A., Nedoma J., Martinek R., Deveci M. (2023). Fundus-DeepNet: Multi-label deep learning classification system for enhanced detection of multiple ocular diseases through data fusion of fundus images. Inf. Fusion.

[2] Goh J.H.L., Ang E., Srinivasan S., Lei X., Loh J., Quek T.C., Xue C., Xu X., Liu Y., Cheng C.-Y. (2024). Comparative analysis of vision transformers and conventional convolutional neural networks in detecting referable diabetic retinopathy. Ophthalmol. Sci..

[3] Teo Z.L., Tham Y.C., Yu M., Chee M.L., Rim T.H., Cheung N., Bikbov M.M., Wang Y.X., Tang Y., Lu Y. (2021). Global Prevalence of Diabetic Retinopathy and Projection of Burden through 2045. Ophthalmology.

[4] Mitry D., Charteris D.G., Fleck B.W., Campbell H., Singh J. (2009). The epidemiology of rhegmatogenous retinal detachment: Geographical variation and clinical associations. Br. J. Ophthalmol..

[5] Holden B.A., Fricke T.R., Wilson D.A., Jong M., Naidoo K.S., Sankaridurg P., Wong T.Y., Naduvilath T., Resnikoff S. (2016). Global prevalence of myopia and high myopia and temporal trends from 2000 through 2050. Ophthalmology.

[6] Ting D.S.W., Cheung C.Y.L., Lim G., Tan G.S.W., Quang N.D., Gan A., Hamzah H., Garcia-Franco R., Yeo I.Y.S., Lee S.Y. (2017). Development and validation of a deep learning system for diabetic retinopathy and related eye diseases using retinal images from multiethnic populations with diabetes. JAMA.

[7] Abràmoff M.D., Folk J.C., Han D.P., Walker J.D., Williams D.F., Russell S.R., Massin P., Cochener B., Gain P., Tang L. (2013). Automated analysis of retinal images for detection of referable diabetic retinopathy. JAMA Ophthalmol..

[8] LeCun Y., Bengio Y., Hinton G. (2015). Deep learning. Nature.

[9] Gulshan V., Peng L., Coram M., Stumpe M.C., Wu D., Narayanaswamy A., Venugopalan S., Widner K., Madams T., Cuadros J. (2016). Development and validation of a deep learning algorithm for detection of diabetic retinopathy in retinal fundus photographs. JAMA.

[10] Ting D.S.W., Pasquale L.R., Peng L., Campbell J.P., Lee A.Y., Raman R., Tan G.S.W., Schmetterer L., Keane P.A., Wong T.Y. (2019). Artificial intelligence and deep learning in ophthalmology. Br. J. Ophthalmol..

[11] Li Z., He Y., Keel S., Meng W., Chang R.T., He M. (2018). Efficacy of a deep learning system for detecting glaucomatous optic neuropathy based on colour fundus photographs. Ophthalmology.

[12] Brown J.M., Campbell J.P., Beers A., Chang K., Ostmo S., Chan R.V.P., Dy J., Erdogmus D., Ioannidis S., Kalpathy-Cramer J. (2018). Automated Diagnosis of Plus Disease in Retinopathy of Prematurity Using Deep Convolutional Neural Networks. JAMA Ophthalmol..

[13] Pead E., Megaw R., Cameron J., Fleming A., Dhillon B., Trucco E., MacGillivray T. (2019). Automated detection of age-related macular degeneration in color fundus photography: A systematic review. Surv. Ophthalmol..

[14] Hemal M.M., Saha S. (2025). Explainable deep learning-based meta-classifier approach for multi-label classification of retinal diseases. Array.

[15] Karthikeyan S., Kumar P.S., Madhusudan R.J., Sundaramoorthy S.K., Namboori P.K.K. (2019). Detection of Multi-Class Retinal Diseases Using Artificial Intelligence: An Expeditious Learning Using Deep CNN with Minimal Data. Biomed. Pharmacol. J..

[16] Shen D., Wu G., Suk H.I. (2017). Deep learning in medical image analysis. Annu. Rev. Biomed. Eng..

[17] Sharmin S., Rashid M.R., Khatun T., Hasan Z., Uddin M.S., Marzia (2024). A dataset of color fundus images for the detection and classification of eye diseases. Data Brief.

[18] Rashid M.R., Sharmin S., Khatun T., Hasan M.Z., Uddin M.S. (2024). Eye Disease Image Dataset. Mendeley Data.

[19] Deng J., Dong W., Socher R., Li L.-J., Li K., Fei-Fei L. ImageNet: A large-scale hierarchical image database. Proceedings of the 2009 IEEE Conference on Computer Vision and Pattern Recognition.

[20] He K., Zhang X., Ren S., Sun J. Deep residual learning for image recognition. Proceedings of the 2016 IEEE Conference on Computer Vision and Pattern Recognition (CVPR).

[21] Tan M., Le Q. (2021). EfficientNetV2: Smaller models and faster training. arXiv.

[22] Redmon J., Divvala S., Girshick R., Farhadi A. You only look once: Unified, real-time object detection. Proceedings of the 2016 IEEE Conference on Computer Vision and Pattern Recognition (CVPR).

[23] Loshchilov I., Hutter F. (2017). Decoupled weight decay regularization. arXiv.

[24] Yang X.L., Yi S.L. (2022). Multi-classification of fundus diseases based on DSRA-CNN. Biomed. Signal Process. Control.

[25] Islam M.T., Imran S.A., Arefeen A., Hasan M., Shahnaz C. Source and camera independent ophthalmic disease recognition from fundus image using neural network. Proceedings of the 2019 IEEE International Conference on Signal Processing, Information, Communication & Systems (SPICSCON).

[26] Deng X., Ding F. Classification of fundus diseases based on meta-data and EB-IRV2 network. Proceedings of the Fourteenth International Conference on Digital Image Processing (ICDIP).

[27] Vali M., Mohammadi M., Zarei N., Samadi M., Atapour-Abarghouei A., Supakontanasan W., Suwan Y., Subramanian P.S., Miller N.R., Kafieh R. (2023). Differentiating glaucomatous optic neuropathy from non-glaucomatous optic neuropathies using deep learning algorithms. Arch. Ophthalmol..

